# New classifications for quantum bioinformatics: Q-bioinformatics, QCt-bioinformatics, QCg-bioinformatics, and QCr-bioinformatics

**DOI:** 10.1093/bib/bbae074

**Published:** 2024-03-05

**Authors:** Majid Mokhtari, Samane Khoshbakht, Kobra Ziyaei, Mohammad Esmaeil Akbari, Sayyed Sajjad Moravveji

**Affiliations:** Department of Bioinformatics, Kish International Campus, University of Tehran, Kish Island, Iran; Department of Bioinformatics, Kish International Campus, University of Tehran, Kish Island, Iran; Duke Molecular Physiology Institute, Duke University School of Medicine-Cardiology, Durham, NC, 27701, USA; Department of Fisheries, Faculty of Natural Resources, University of Tehran, Karaj, Iran; Cancer Research Center, Shahid Beheshti University of Medical Sciences, Tehran, Iran; Department of Bioinformatics, Kish International Campus, University of Tehran, Kish Island, Iran

**Keywords:** Quantum mechanics, Quantum biology, Quantum bioinformatics

## Abstract

Bioinformatics has revolutionized biology and medicine by using computational methods to analyze and interpret biological data. Quantum mechanics has recently emerged as a promising tool for the analysis of biological systems, leading to the development of quantum bioinformatics. This new field employs the principles of quantum mechanics, quantum algorithms, and quantum computing to solve complex problems in molecular biology, drug design, and protein folding. However, the intersection of bioinformatics, biology, and quantum mechanics presents unique challenges. One significant challenge is the possibility of confusion among scientists between quantum bioinformatics and quantum biology, which have similar goals and concepts. Additionally, the diverse calculations in each field make it difficult to establish boundaries and identify purely quantum effects from other factors that may affect biological processes. This review provides an overview of the concepts of quantum biology and quantum mechanics and their intersection in quantum bioinformatics. We examine the challenges and unique features of this field and propose a classification of quantum bioinformatics to promote interdisciplinary collaboration and accelerate progress. By unlocking the full potential of quantum bioinformatics, this review aims to contribute to our understanding of quantum mechanics in biological systems.

## INTRODUCTION

Bioinformatics is a discipline that employs computational methods to analyze and interpret biological data [1, 2]. It is an interdisciplinary field that merges biology, computer science, and mathematics, and has played a crucial role in advancing our understanding of biological processes [3]. In recent years, quantum mechanics has emerged as a tool for analyzing biological systems, and the intersection of bioinformatics, biology, and quantum mechanics has led to the emergence of quantum bioinformatics [4].

Quantum bioinformatics is a relatively new field that seeks to apply the principles of quantum mechanics to the analysis of biological systems. It leverages quantum algorithms and quantum computing to solve complex problems in molecular biology, drug design, and protein folding, and has the potential to revolutionize our understanding of biological processes [5].

However, there are several challenges associated with the intersection of bioinformatics, biology, and quantum mechanics. One major challenge is the possibility of confusion among scientists, as quantum biology and quantum bioinformatics share similar goals and deal with similar concepts. Additionally, it can be challenging to separate purely quantum effects from other factors that may affect biological processes, and there is a need to classify different subsets of quantum bioinformatics to fully understand the field.

**Figure 1:**
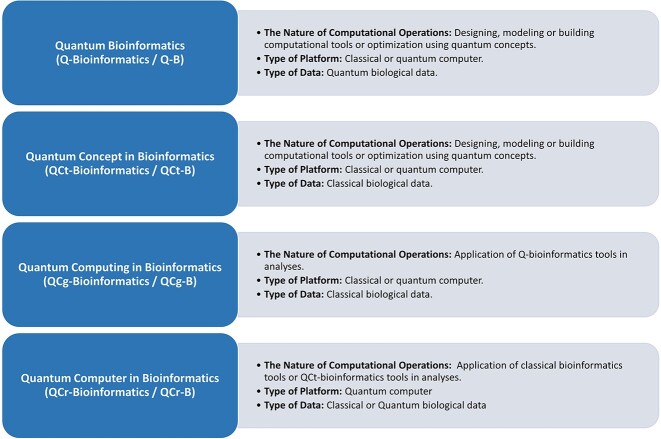
Diverse Classes in Quantum Bioinformatics. This figure illuminates multiple classes within the realm of Quantum Bioinformatics. It delineates Quantum Bioinformatics (Q-Bioinformatics / Q-B), Quantum Concept in Bioinformatics (QCt-Bioinformatics / QCt-B), Quantum Computing in Bioinformatics (QCg-Bioinformatics / QCg-B), and Quantum Computer in Bioinformatics (QCr-Bioinformatics / QCr-B). Each class is characterized by specific computational operations, platform types, and data categories, providing a comprehensive snapshot of the diverse applications and methodologies within the field.

To address these challenges, this review provides an overview of the concepts of bioinformatics and quantum mechanics and their intersection in quantum bioinformatics. We discuss the unique features of quantum bioinformatics, such as quantum algorithms and quantum computing, and examine the challenges in analyzing and interpreting quantum biological data. Finally, we provide a standard classification and usable terminology for quantum bioinformatics to promote interdisciplinary collaborations and accelerate progress in the field (see Figure 1).

In the following sections, we examine the principles and concepts of quantum mechanics, provide an overview of quantum biology, and discuss the emergence of quantum bioinformatics. We also examine the challenges associated with quantum bioinformatics, including the difficulty of separating purely quantum effects from other factors and the need to classify different subsets of the field. By providing a comprehensive understanding of quantum bioinformatics, we hope to unlock the full potential of this field and make strides in understanding quantum mechanics in biological systems.

## QUANTUM CONCEPTS IN BIOLOGY

The current understanding of biology has been successful in applying classical models to living systems, but often disregards subtle quantum effects on a molecular scale. The concept of ‘function’ in biology includes processes such as vision, photosynthesis, DNA replication, and brain processing, all of which are subject to the laws of quantum mechanics. However, the differences between a classical approximation and a quantum-mechanical model are typically considered insignificant in these situations. The field of quantum biology has emerged to explore the connection between quantum mechanics and biological processes that classical physics cannot fully explain [4].

Quantum biology is the application of quantum theory to biological phenomena that classical physics cannot adequately explain. This field has gained prominence in recent years, with investigations into phenomena such as vision, photosynthesis, and avian navigation. It has been hypothesized that living organisms use principles of quantum informatics and that the missing laws of life are those of chance and probability in the quantum world [6–9].

While the study of quantum effects in complex biological systems has existed since the beginning of quantum mechanics, it has only recently gained attention as a testable concept [6]. There is increasing evidence that certain biological processes utilize non-trivial aspects of quantum mechanics, including long-lived quantum coherence, superposition, quantum tunneling, and quantum entanglement [10, 11]. Examples of these phenomena in biology include the transport of exciton energy during photosynthesis [12–15], the tunneling of electrons and protons during enzyme catalysis [16, 17], and the potential role of quantum tunneling in olfaction [18] and mutation [19].

**Figure 2:**
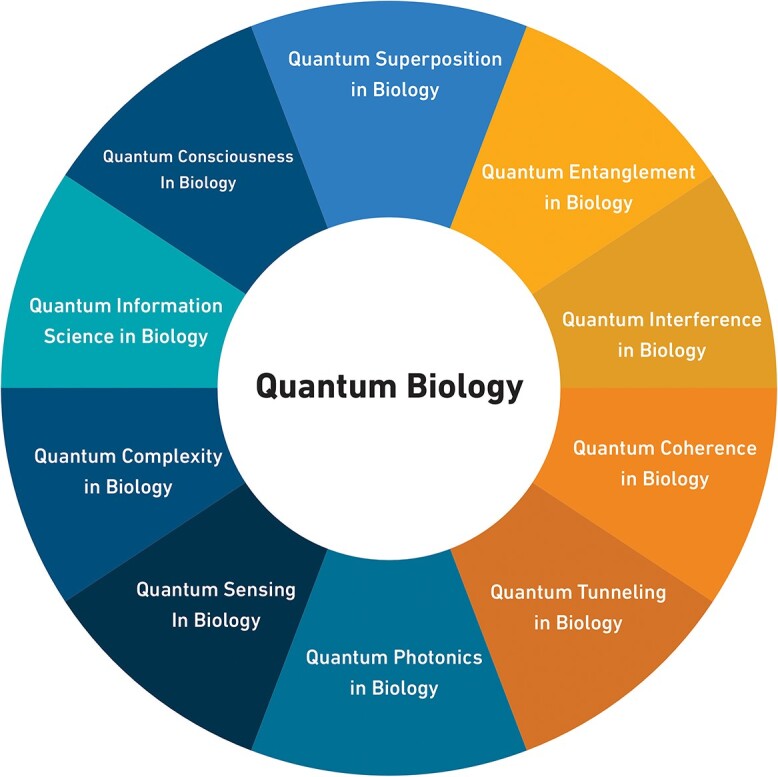
Quantum Concepts in Biology. In Figure 2, quantum biology is organized in accordance with the principles of quantum mechanics that can produce biological consequences.

While some have suggested a connection between quantum coherence and consciousness, this idea is not widely accepted in the neurobiology community [20]. Nevertheless, the investigation of quantum biology has grown in the past two decades, and researchers are using numerical methods to solve relevant Schrödinger equations and explain various biological phenomena. Therefore, in the following, we will examine how to use the features of quantum mechanics to explain various biological phenomena (see Figure 2).

### Quantum superposition in biology

Quantum superposition is a fundamental concept in quantum mechanics, allowing a quantum bit, or qubit, to exist in multiple states simultaneously until measured. Unlike classical bits, which can only be in one of two states, the ability of a quantum computer to utilize superposition is its most critical feature, enabling the computation of many possible solutions simultaneously [21]. The state of a qubit is described by a superposition of basis states *|0 >* and *|1>*, represented as *|ψ > = α |0 > + β |1>*, where α and *β* are complex coefficients known as amplitudes, reflecting the probability of the qubit being in state *|0 >* or *|1>*, respectively. These amplitudes must satisfy the normalization condition *|α|^2 + |β|^2 = 1* to ensure the total probability of finding the qubit in either state is *1* [22–24]. Quantum superposition’s significance lies in its capacity to solve problems in parallel, exemplified in search algorithms. Classical computing requires sequential search through a database, while quantum computing can use superposition to explore all possible items simultaneously, greatly accelerating the search process [25].

Measurement is a critical aspect of quantum mechanics as it allows obtaining information about the system’s state. However, it destroys the superposition of a qubit, causing it to collapse to one of the two possible classical bit states. When designing quantum algorithms, the alteration of the system’s state due to measurement must be taken into account [26]. Quantum superposition’s importance extends to quantum biology, where it serves to explain various biological systems’ remarkable organization despite external noise or interference. In photosynthesis, energy transfer between molecules is efficiently accomplished through superposition, distributing energy across multiple states or pathways, minimizing energy loss even in the presence of perturbations like temperature changes or electromagnetic fluctuations [27, 28]. In bird navigation, some species rely on superposition to navigate using the Earth’s magnetic field. Magnetic sensors existing in multiple states simultaneously enable the bird to obtain information about the magnetic field’s direction and strength from multiple sources at once, processed by the brain to determine its location and orientation [27, 29, 30]. Similarly, in olfaction, the sense of smell, superposition is suggested to play a role, with odor molecules binding to receptors in multiple states, allowing the detection of multiple odor molecules at once, enabling the brain to identify specific odors [31–33].

Beyond these examples, superposition is speculated to have implications in memory storage, synaptic transmission, and gene regulation, although further research is needed to fully understand its role in these processes [34–37]. In conclusion, superposition is a powerful and versatile concept in quantum mechanics that explains puzzling phenomena in the quantum world. Its application in quantum biology has led to exciting insights into how biological systems maintain their functionality despite external disturbances. Though the relationship between superposition and quantum biology requires further exploration, its potential to advance our understanding of the principles governing biological systems is promising [27, 29, 31–34].

### Quantum entanglement in biology

Quantum entanglement is a remarkable phenomenon in quantum mechanics where two or more qubits become correlated in such a way that their combined state cannot be expressed as a simple product of their individual states [38].

This entanglement is described by a mathematical concept involving tensor products of state vectors, represented as *|ψ > = 1/√2 (|a > ⊗ |b > + |b > ⊗ |a>)* [39, 40], where the symbol *⊗* denotes the tensor product. The resulting state is a superposition of *|a > ⊗ |b >* and *|b > ⊗ |a>*, which cannot be factored into individual states.

Entanglement finds applications in quantum technologies, with quantum teleportation being a significant one. This protocol enables the teleportation of an unknown quantum state from one location to another using entanglement and classical communication [41, 42]. By utilizing entanglement, the state can be teleported without physically moving the qubit itself.

The importance of entanglement and superposition in quantum computing lies in their ability to provide an exponential speedup for specific computational tasks. Superposition allows quantum computers to perform computations on multiple inputs simultaneously, while entanglement enables the manipulation of vast amounts of information with minimal operations. This powerful combination makes quantum computing a valuable tool for solving problems that are beyond the capabilities of classical computers.

In the field of quantum biology, entanglement has intriguing implications [38]. It has been proposed to play a crucial role in energy transfer during photosynthesis. The process involves entanglement, where energy from one molecule becomes correlated with the state of another, allowing efficient energy transfer over long distances, even in noisy environments [28, 43].

Additionally, quantum entanglement is vital for the avian compass’s radical pair mechanism. An entangled state is produced through electron photoexcitation and translocation, generating required spin correlations. This causes electrons to fluctuate between singlet and triplet states, influencing sensitivity to magnetic fields during avian navigation [44].

Entanglement’s involvement in olfaction suggests that odor molecules can be correlated with receptors on olfactory neurons, enabling detection even when the molecules are spatially separated [45].

Furthermore, entanglement is considered essential for quantum memory storage. Information is stored in a highly organized and correlated state among quantum particles like atoms or ions. This entangled state ensures that the information of one particle is correlated with another, allowing retrieval and processing despite large distances and the presence of external noise or interference [46–48].

In conclusion, quantum entanglement is a captivating concept with broad implications for quantum computing and biology. Its role in energy transfer, olfaction, and quantum memory storage opens new perspectives on how biological systems remain functional in the presence of noise and interference. While much research is still required to fully understand the relationship between entanglement and quantum biology, it offers promising advancements in understanding the fundamental principles governing biological systems.

### Quantum interference in biology

Interference is a fundamental phenomenon in quantum mechanics, where two or more waves interact, resulting in constructive or destructive interference. In quantum systems, interference plays a vital role in measurements and system behavior. When probability amplitudes between quantum states are added or subtracted, quantum interference occurs, leading to distinct outcomes [49, 50].

A classic example of quantum interference is the Hadamard gate, a single-qubit gate. Applying the Hadamard gate to the input state |0 > results in an equal superposition of *|0 >* and *|1>*:


$$ H\left|0>=\left(\left|0>+\right|1>\right)/\sqrt{2}\right. $$

Further application of the Hadamard gate leads to destructive interference:


$$ H\left(H|0>\right)=\left(\left|0>-\right|1>\right)/\surd 2. $$

Here, the probability amplitudes cancel each other out, resulting in an equal superposition of *|0 > and |1 >* [22].

The double-slit experiment is another example of quantum interference. Particles passing through two slits create an interference pattern, demonstrating the wave-particle duality of quantum systems [51, 52].

Interference is crucial in quantum mechanics as it allows quantum systems to exhibit behaviors impossible for classical systems. It plays a pivotal role in quantum algorithms and enables the development of quantum technologies, including cryptography, sensing, and computing.

In the field of biology, quantum interference is believed to have significant implications in various processes. Photosynthesis, the conversion of light energy into chemical energy, benefits from quantum interference in its initial stages. Quantum interference between different energy transfer pathways is thought to enhance photosynthetic efficiency, surpassing classical predictions [53].

Enzymes, biological catalysts, are influenced by quantum interference, particularly in the rate of enzymatic reactions. Quantum interference can enhance proton transfer rates during reactions by interfering with the pathways involved [28].

Sensory perception, the ability to detect and respond to external stimuli, may also involve quantum interference. The transport of ions across cell membranes, influenced by quantum interference, could affect the sensitivity of nerve cells to external stimuli [45].

Although the understanding of the relationship between quantum interference and biology is still in its early stages, the potential applications are vast. Insights gained from studying quantum interference in photosynthesis may contribute to the development of more efficient solar cells. Understanding the impact of quantum interference on enzymatic reactions could lead to the design of more effective drugs. Exploring how quantum interference influences sensory perception opens up new frontiers in both quantum physics and biology, holding promising possibilities for advancements in both fields.

### Quantum coherence in biology

Quantum coherence is a fundamental property of quantum systems, enabling them to exist in multiple states simultaneously and maintain a well-defined phase relationship between these states [54, 55]. This phenomenon is crucial in various fields, including physics, chemistry, and biology, as it underlies essential phenomena such as superposition, entanglement, and quantum tunneling [29, 56].

Mathematically, quantum coherence is described through wave functions, denoted by *Ψ*, which encode the probability amplitudes of different quantum states [55]. The wave function can be represented as *Ψ = ∑ c_i |ψ_i⟩*, where *c_i* represents the complex probability amplitude of the *i-th* state, and *|ψ_i⟩* is the corresponding quantum state. The complex probability amplitudes signify the phase relationships between the states, and the sum of all possible states ensures the total probability is normalized to unity.

The Schrödinger equation is an essential formula related to quantum coherence, describing the time evolution of a quantum system [57]. It is given by ${{i}\hbar\ \partial\Psi}/{\partial t =H \Psi} $, where $\hbar $ is the reduced Planck constant, *H* is the Hamiltonian operator describing the system’s energy, and t represents time. The Schrödinger equation demonstrates that the wave function evolves deterministically over time, while the phase relationship between different states may change based on the system’s energy.

Another vital formula related to quantum coherence is the coherence time (*T_2*). This measure indicates how long a quantum system can maintain coherence before being affected by external disturbances. Techniques such as quantum interferometry and NMR spectroscopy are used to estimate the coherence time. The coherence time is of great significance in quantum computing and quantum communication, as it impacts the system’s ability to perform complex quantum operations and store quantum information.

Quantum coherence plays a critical role in biological systems, particularly in excitation energy transfer and charge transfer during photosynthesis [29, 58]. This process involves capturing sunlight by photosynthetic pigment-protein complexes and delivering the energy as electronic excitation to the reaction center for charge separation, powering biochemistry. Coherence dynamics and quantum superposition are essential in achieving the high quantum efficiency observed in natural light harvesting.

Excitonic coherence, resulting from the superposition of exciton states, is another quantum phenomenon at work in photosynthesis [56, 59]. The coherence between exciton states is necessary for coherent wave-like dynamics. It is the term used to describe coherence between exciton states.

Coherence also plays a role in avian navigation, where birds use the avian compass to determine the Earth’s magnetic field during migration [38, 60, 61]. Coherence allows magnetic information to be transferred between molecules in the avian eye, even in the presence of environmental noise or interference.

Furthermore, coherence may be involved in the sense of smell, where odor molecules potentially bind to receptors on the surface of olfactory neurons in a highly organized and correlated state [62]. This coherence enables receptors to detect the presence of odor molecules even when separated by a large distance, such as in the air.

The study of coherence in biological systems holds promise for advancing our understanding of how these systems maintain highly organized states despite external influences [28]. Applications of coherence in energy transfer, avian navigation, and olfaction offer new perspectives on maintaining consistency and organization. While further research is needed, the role of coherence in fundamental processes is evident, with potential impacts on various fields, including medicine and technology.

### Quantum Tunneling in biology

Quantum tunneling is a remarkable phenomenon in which particles can pass through potential barriers, defying classical expectations [63, 64]. In various fields, including solid-state physics, chemistry, and nuclear physics, understanding quantum tunneling holds significant implications [65].

The probability of quantum tunneling can be quantified using the transmission coefficient (*T*), given by the formula *T = e^(−2γ)*. Here, *γ* represents the imaginary part of the wave vector in the barrier region. This formula demonstrates the exponential decrease in the probability of tunneling as the barrier thickness increases.

The wave function describing a particle tunneling through a potential barrier is given by *Ψ(x) = A e^(ik₁x) + B e^(−ik₁x)*. Here, *A* and *B* are coefficients for the incident and reflected waves, respectively. The wave vector in the incident region is denoted as *k₁*, and *x* represents the distance along the direction of motion. This formula illustrates that the wave function of a tunneling particle consists of both incident and reflected components.

Quantum tunneling finds relevance in solid-state physics for comprehending the behavior of semiconductors and superconductors, as well as in nuclear physics for understanding the decay of unstable nuclei. In chemistry, it is crucial for elucidating the rates of chemical reactions, particularly those involving hydrogen transfer. Moreover, technologies such as scanning tunneling microscopy rely on quantum tunneling to enable atomic-level surface imaging. Understanding the principles of quantum tunneling is vital for the development of quantum-based technologies like computing and cryptography [65].

In the biological realm, quantum tunneling plays essential roles in enzymatic processes [28, 66] and the transport of protons and electrons across biological membranes [67]. Enzymes act as catalysts in living organisms, facilitating chemical reactions that involve electron or proton transfer. Quantum tunneling aids in the transfer of these particles through energy barriers that would impede reaction rates [68]. For instance, cytochrome c oxidase, an enzyme involved in cellular respiration, utilizes quantum tunneling to efficiently transfer electrons between molecules, enabling energy production [69].

Beyond enzymes, quantum tunneling is crucial in proton and electron transport across biological membranes [67]. Proton transport is vital for various biological processes, including ATP generation, the cell’s energy currency [70, 71]. In photosynthesis, quantum tunneling is believed to contribute to electron transfer across the photosynthetic membrane, facilitating the conversion of light energy into chemical energy [72].

Furthermore, quantum tunneling has been proposed as a mechanism for olfactory receptors’ ability to detect odor molecules. The hypothesis suggests that odor molecules can tunnel through the mucus layer in the nose, interacting with olfactory receptors and triggering neural responses [73]. Although still under investigation, this theory highlights the potential involvement of quantum phenomena in biological systems.

Recent research has also proposed a role for quantum tunneling in DNA replication [74]. Scientists at the University of California, Berkeley, suggested that quantum tunneling may explain how DNA polymerase, an enzyme responsible for DNA replication, accurately reads and replicates the genetic code. The concept posits that quantum tunneling allows the enzyme to explore multiple conformations of the DNA molecule, enhancing its chances of identifying the correct one [75].

In conclusion, quantum tunneling holds substantial significance in biology, manifesting in enzymatic processes, proton and electron transport across biological membranes, and potentially even DNA replication. Exploring the implications of quantum tunneling in biological systems presents new avenues for research and the potential development of technologies and medical treatments.

### Quantum photonics in biology

Quantum photonics is a rapidly growing field that explores the behavior of light and its interaction with matter from a quantum mechanical perspective. It has the potential to revolutionize communication, sensing, and computing technologies by leveraging the unique properties of quantum mechanics [76]. Photons, the basic units of light, exhibit wave-particle duality, superposition, and entanglement, which are governed by the laws of quantum mechanics.

In quantum photonics, important formulas include Planck’s law, which relates the energy of a photon to its frequency using the equation *E = hf* [77]. The wave function, represented by *Ψ(x, t)*, describes the spatial and temporal behavior of a photon and is given by *Ae^(i(kx - ωt))*, where *A* is the amplitude, k is the wave vector, *x* is the position, *ω* is the frequency, and t is the time. The Schrödinger equation, *i*$ \hbar $*(∂Ψ/∂t) = HΨ*, governs the time evolution of the wave function, where $ \hbar $ is the reduced Planck’s constant, *H* is the Hamiltonian operator, and *Ψ* is the wave function [78].

Understanding quantum photonics is crucial for developing technologies that surpass classical optics. Quantum cryptography, for example, exploits entangled photons to enable secure communication that cannot be intercepted without detection [79]. Quantum sensing can achieve unprecedented levels of accuracy in detecting environmental changes, such as magnetic fields or temperature. Quantum computing harnesses the manipulation of individual photons to perform computations exponentially faster than classical computers [80].

Moreover, quantum photonics finds applications in studying biological systems at the nanoscale [80, 81]. Researchers employ quantum photonics techniques to investigate the interactions between light and biomolecules, such as proteins and DNA, with unprecedented detail [82]. Techniques like coherent anti-Stokes Raman scattering (CARS) microscopy utilize ultrafast laser pulses to visualize living cells and tissues, revealing the distribution and composition of biomolecules [83, 84]. Quantum photonics also aids in the development of highly sensitive and specific biosensors. Plasmonic nanoparticles, for instance, enable the detection of specific DNA sequences with high accuracy [85, 86].

Furthermore, quantum photonics holds potential in the field of optogenetics, which employs light to control specific cell activity in the brain [87, 88]. Quantum photonics techniques can enhance the precision and efficiency of optogenetics tools. Two-photon optogenetics, using ultrafast laser pulses, allows for precise control of neuron activity with high spatial and temporal resolution [89, 90].

In summary, quantum photonics is an evolving field that exploits the quantum behavior of light to advance technologies and explore biological systems. It enables the study of biomolecules, the development of sensitive biosensors, and the refinement of optogenetics tools. Continued advancements in quantum photonics are expected to lead to further exciting applications in biology and other fields.

### Quantum sensing in biology

Quantum sensing is a cutting-edge field that leverages the principles of quantum mechanics to achieve incredibly precise measurements of physical quantities. By manipulating and observing quantum systems, valuable information about the surrounding environment can be extracted [91]. Quantum sensors find applications in diverse fields like navigation, medical diagnostics, and environmental monitoring [92–94].

Magnetic field measurement stands as one of the prominent applications of quantum sensing [95]. This involves detecting the spin of electrons or nuclei in a magnetic field using techniques like nuclear magnetic resonance (NMR) and electron spin resonance (ESR) [96]. The sensor’s sensitivity relies on the coherence time of the quantum system utilized for detecting the physical quantity [97]. Coherence time refers to the duration that a quantum system maintains its coherence, i.e., its ability to exist in a superposition of states.

To describe the time evolution of magnetization in a magnetic field, the Bloch equation plays a crucial role [98]. The equation is given by dM/dt = γ * (M x B) - M/T2, where *M* represents magnetization, *B* is the magnetic field, *γ* denotes the gyromagnetic ratio, and *T2* stands for the spin–spin relaxation time.

An important principle that affects quantum sensing is the Heisenberg uncertainty principle [99, 100]. It posits that the simultaneous reduction of uncertainty in two complementary variables, like position and momentum, is not possible, setting fundamental limits on measurement sensitivity.

Quantum sensing finds promising applications in biology, where it enables the measurement and monitoring of biological processes with remarkable precision and sensitivity [57]. One area where it proves useful is in magnetic sensing [101]. Biological processes often involve magnetic field interactions, such as bird migration and marine animal navigation. Quantum sensors based on nitrogen-vacancy (NV) centers in diamond have shown high sensitivity in detecting biological magnetic fields [102, 103]. They have been employed to measure magnetic fields produced by neural activity in mice brains [104] and study the magnetic properties of bacteria [105].

Moreover, quantum sensing holds potential in biological imaging, overcoming the limitations of traditional techniques like X-rays and MRI. NV centers and other quantum systems facilitate high-resolution imaging at the nanoscale level [106, 107]. They allow the imaging of individual protein structures [108, 109] and the distribution of ions in cells [110].

Beyond magnetic sensing and imaging, quantum sensing finds applications in biosensing and biomolecule detection [111, 112]. Quantum sensors can be designed to detect specific molecules or biomarkers with exceptional sensitivity and selectivity, offering opportunities for revolutionizing medical diagnostics and personalized medicine [113–115].

Furthermore, quantum sensing offers a unique avenue to explore the fundamental physics of biological systems. Processes like photosynthesis and enzymatic reactions involve quantum effects such as coherence and tunneling [102]. With sensitive quantum sensors, researchers can delve into these quantum phenomena and gain insights into their contributions to the overall biological functions.

In conclusion, quantum sensing is a rapidly evolving field with immense potential for applications in biology. It enables precise and sensitive measurements of biological processes, leading to a deeper understanding of fundamental physics in biology and opening up avenues for advanced medical diagnostics and personalized medicine. As technology progresses, the impact of quantum sensing on various disciplines is expected to be revolutionary.

### Quantum complexity in biology

Quantum complexity theory, a branch of theoretical computer science, delves into the computational complexity of quantum algorithms and their resource requirements [116]. Central to this field are quantum circuits, sequences of quantum gates acting on qubits, the basic units of information in quantum computers [117]. Quantum oracles, black boxes providing access to unknown functions, also play a key role, enabling algorithms to query and learn about these functions [118].

In quantum complexity theory, significant algorithms and formulas stand out. The Deutsch-Jozsa algorithm addresses the Deutsch-Jozsa problem, determining whether a function *f(x)* is constant or balanced. Utilizing a single query to the quantum oracle, this algorithm runs in *O(1)* time on a quantum computer, far more efficient than the *O(2^n)* time on a classical computer [118, 119]. Grover’s algorithm, designed to solve the search problem in an unsorted database, uses multiple queries to the quantum oracle, running in *O(sqrt(N))* time on a quantum computer compared to *O(N)* on a classical computer [120, 121]. Shor’s algorithm, a pivotal factorization solver, finds the prime factors of a large composite number using multiple queries to the quantum oracle. It runs in *O((log N)^3)* time on a quantum computer, a vast improvement compared to the *O(exp((1/3)(log N)^(1/3)(log log N)^(2/3)))* time on a classical computer [122, 123].

Beyond computer science, quantum complexity manifests in biology, influencing several crucial biological processes. Notably, photosynthesis employs quantum coherence to capture light energy efficiently. In this process, chromophores transfer energy to reaction centers through entangled quantum states, increasing the overall efficiency of the energy transfer [6, 124].

The behavior of enzymes, which catalyze chemical reactions in living organisms, also demonstrates quantum complexity [125, 126]. The interactions between atoms and molecules in enzymes are governed by quantum mechanics, making it difficult to fully understand their molecular-level workings [125, 127]. Recent studies suggest that enzymes utilize quantum tunneling to catalyze otherwise improbable reactions [128]. Quantum tunneling allows particles to pass through energy barriers classically considered insurmountable, enabling enzymes to catalyze reactions involving the transfer of protons or electrons [65].

Quantum complexity extends its influence to the human brain as well. Neurons, the cells of the nervous system, communicate through neurotransmitter release, a process dependent on the quantum mechanical behavior of calcium ions. The intricate nature of this process defies classical simulation, indicating that quantum coherence and entanglement may contribute to the efficient communication between neurons [129, 130].

In conclusion, quantum complexity theory, while integral to the analysis of quantum algorithms, also finds significant application in biology. The efficient energy transfer in photosynthesis, quantum effects in enzyme catalysis, and quantum behavior in neuron communication demonstrate the far-reaching impact of quantum complexity in understanding fundamental biological processes. The burgeoning field of quantum biology continues to shed light on the intricate workings of life at the quantum level.

### Quantum information science in biology

Quantum information science (QIS) merges quantum mechanics principles with information processing, revolutionizing fields such as communication, computation, and cryptography. Unlike classical information, quantum information relies on qubits, which can exist in superpositions and become entangled with other qubits [131]. The qubit state vector, denoted as *|ψ⟩*, represents a linear combination of basis states *|0⟩* and *|1⟩*, characterized by complex coefficients *α* and *β* [132]: *|ψ⟩ = α|0⟩ + β|1⟩.*

The density matrix, symbolized as *ρ*, describes the probability of finding a qubit in a specific state and is calculated from the state vector [133]: *ρ = |ψ⟩⟨ψ|.*

Entanglement plays a crucial role in quantum information, enabling correlated states beyond classical explanations and facilitating unique communication and computation capabilities [134].

The potential of quantum information is far-reaching, transforming numerous scientific and technological domains. Quantum computers have the ability to outperform classical computers in specific calculations, impacting fields like cryptography and drug discovery [135, 136]. Quantum communication offers secure transmission immune to hacking and eavesdropping [137]. Additionally, quantum sensors possess unparalleled sensitivity for detecting signals in medical imaging and environmental monitoring [138, 139].

The interdisciplinary nature of QIS presents promising applications in biology [138]. Quantum computing, for instance, can tackle complex biological problems like protein folding and drug design more efficiently than classical computers. By harnessing quantum principles, quantum computers can operate on multiple qubits simultaneously, exploring numerous potential solutions at once [140, 141].

Furthermore, QIS finds relevance in studying biomolecules, where quantum mechanics fundamentally shapes their behavior. By delving into the quantum properties of biomolecules like DNA and proteins, researchers gain deeper insights into their functionality and manipulative potential [75].

Quantum communication emerges as a valuable tool in biology, offering inherent security by transmitting information through quantum states. Any attempt to measure or intercept a quantum state alters or destroys it, notifying the sender and receiver of potential eavesdroppers. This characteristic makes quantum communication well-suited for transmitting sensitive biological information without the risk of interception, such as patient health records [142].

Although the relationship between QIS and biology is in its early stages, their potential synergy presents vast research opportunities. Leveraging the principles of quantum mechanics, QIS can revolutionize the study and manipulation of biological systems. Novel tools and techniques can be developed to understand and manipulate biomolecules, fostering breakthroughs in drug design and synthetic biology [138]. By integrating QIS with the intricacies of biological systems, scientists can unravel the mysteries of quantum biology, paving the way for transformative advancements in our understanding of life.

### Quantum consciousness in biology

Over the past several years, terms like ‘quantum consciousness’ and ‘cosmic consciousness’ have been misused for commercial gain and mistakenly associated with new forms of mysticism. This proliferation of false information has prompted us to be cautious in revising the content of these sections. It’s crucial to remember that the scientific research mentioned is rooted in the principles of quantum physics and its findings should not be extended to encompass any mystical subjects outside of what is explicitly stated.

The concept of quantum consciousness is a hypothesis that suggests that quantum mechanics plays a role in the workings of the human brain and consciousness. While there is no scientific evidence to support this idea, it has gained some attention in the field of neuroscience and philosophy. Understanding the relationship between quantum consciousness and biology involves exploring how quantum mechanics might interact with biological processes in the brain [143, 144].

The human brain is made up of cells called neurons, which communicate with each other through electrical and chemical signals. These signals allow the brain to process information and generate conscious experience [145]. According to the quantum consciousness hypothesis, the brain’s ability to generate consciousness may be related to the behavior of subatomic particles within neurons [146].

Quantum mechanics describes the behavior of subatomic particles, such as electrons and photons. These particles exist in a state of superposition, meaning that they can exist in multiple states simultaneously. In addition, quantum particles can become entangled, meaning that their properties become linked even when separated by large distances [4, 22].

Proponents of the quantum consciousness hypothesis suggest that these quantum phenomena may play a role in the workings of the brain [147–149]. For example, they suggest that superposition [150, 151] and entanglement [152] may allow neurons to process information more efficiently and generate conscious experience. However, there is currently no scientific evidence to support these claims.

Despite the lack of evidence, researchers have explored the potential links between quantum mechanics and biological processes in the brain. For example, studies have suggested that certain proteins in the brain may be able to exist in a state of quantum coherence, allowing them to process information in a way that is not possible with classical mechanics [153, 154].

Other researchers have suggested that the phenomenon of quantum tunneling, in which particles can pass through barriers that they would not be able to overcome using classical mechanics, may play a role in the brain’s ability to generate conscious experience [67, 155].

Overall, the relationship between quantum consciousness and biology remains a topic of debate and speculation. While some researchers have explored potential links between quantum mechanics and biological processes in the brain, there is currently no scientific evidence to support the idea that quantum mechanics plays a significant role in human consciousness. Nonetheless, the exploration of this hypothesis may continue to drive research into the workings of the brain and consciousness.

## QUANTUM CONCEPTS IN BIOINFORMATICS AND ITS CLASSIFICATION

Bioinformatics is a multidisciplinary field that combines biology, computer science, and mathematics to analyze and interpret biological data, such as DNA and protein sequences. Its aim is to gain insights into biological processes [1–3].

Quantum through the use of computational tools and techniques. On the other hand, quantum bioinformatics is an emerging field that applies principles from quantum mechanics to the analysis of biological systems. It utilizes quantum algorithms and quantum computing to solve complex problems in molecular biology, drug design, and protein folding. Quantum bioinformatics takes advantage of the unique properties of quantum mechanics, such as superposition and entanglement, to enhance computational approaches in biological research [5].

While both bioinformatics and quantum bioinformatics share the common goal of analyzing and interpreting biological data, they differ in their approaches and tools. Bioinformatics relies on classical computing methods, whereas quantum bioinformatics employs quantum algorithms and quantum computing. Quantum biology is a broader field that explores the role of quantum mechanics in biological systems, encompassing phenomena like photosynthesis, olfaction, and magnetoreception. It seeks to understand how these processes occur and how they can be harnessed for various applications [4, 8].

There is significant overlap between quantum biology and quantum bioinformatics as they both investigate the role of quantum mechanics in biological systems. However, this overlap can create challenges, particularly in terms of confusion among scientists. Since these fields share similar goals and concepts, it can be difficult to differentiate between them, leading to confusion and hindered progress.

Another challenge lies in distinguishing purely quantum effects from other factors influencing biological processes. In quantum biology, it can be challenging to determine whether a phenomenon is solely attributable to quantum effects or if other chemical interactions are at play. Similarly, in quantum bioinformatics, differentiating between computational approaches leveraging quantum effects and those utilizing classical computing methods can be difficult.

To address these challenges, it is crucial to establish clear definitions and standardized terminology for quantum biology and quantum bioinformatics. Additionally, interdisciplinary collaborations among researchers from various fields can facilitate overcoming the obstacles associated with these disciplines. By doing so, we can unlock the full potential of quantum biology and quantum bioinformatics and make significant progress in understanding the role of quantum mechanics in biological systems.

**Figure 3:**
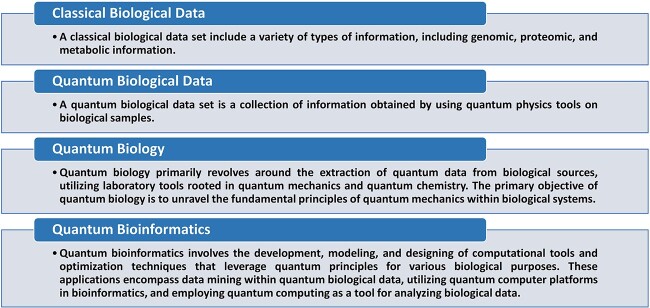
Exploring Quantum Biology and Bioinformatics. This figure provides a concise overview of key concepts in the realm of biological data, quantum biology, and quantum bioinformatics. It illustrates the shift from classical biological data to quantum biological data and highlights the objectives and applications of quantum biology and bioinformatics in leveraging quantum principles for biological research.

When classifying bioinformatics based on quantum concepts, it is important to consider the diverse computational processes employed in each field. Bioinformatics computations typically involve data mining of extracted biological data using classical computing methods. In contrast, quantum biological data is obtained through quantum mechanical methods, and quantum bioinformatics calculations are used for more complex analyses after extracting quantum data. Quantum mechanics, quantum biology, and quantum bioinformatics all require extensive use of computational tools. However, due to the diverse computational processes used in each field, it is difficult to identify the boundaries and limitations of quantum bioinformatics computational. While numerical methods and simulations are commonly used in quantum mechanics and biology, new computational methods such as quantum machine learning and quantum-enhanced algorithms are being developed in quantum bioinformatics. In general, quantum bioinformatics involves the development, modeling, and designing of computational tools and optimization techniques that leverage quantum principles for various biological purposes. These applications encompass data mining within quantum biological data, utilizing quantum computer platforms in bioinformatics, and employing quantum computing as a tool for analyzing biological data. In contrast, quantum biology primarily revolves around the extraction of quantum data from biological sources, utilizing laboratory tools rooted in quantum mechanics and quantum chemistry. The primary objective of quantum biology is to unravel the fundamental principles of quantum mechanics within biological systems. Conversely, quantum bioinformatics specifically concentrates on the utilization of quantum computing methods for the analysis of biological data (see Figure 3).

Biological data can encompass various types of data, such as genomic, proteomic, and metabolic data [156, 157]. In contrast, quantum data can be classified based on the nature of the data and the instruments used to obtain it. For instance, quantum state data describes the state of a quantum system and can be obtained from instruments such as quantum state tomography machines or quantum state discriminators [158, 159]. Quantum measurement data describes the result of a measurement on a quantum system and can be obtained from instruments such as quantum photodetectors [160] or superconducting qubit readout devices [161, 162]. Quantum communication data describes the transmission of quantum states between different locations and can be obtained from instruments such as quantum communication systems [163] or quantum key distribution devices [164, 165]. Quantum computing data describes the processing of quantum information and can be obtained from instruments such as quantum computers [166] or quantum simulators [167]. Quantum sensing data describes the measurement of physical quantities using quantum systems and can be obtained from instruments such as quantum sensors [168] or atomic clocks [169]. Lastly, quantum imaging data describes the visual representation of quantum systems and can be obtained from instruments such as quantum microscopes [170, 171] or quantum cameras [172]. Each of these data types corresponds to different aspects of quantum systems and can be obtained using specific scientific instruments. A quantum biology data set is a collection of information obtained by using quantum physics tools on biological samples (see Figure 3).

The main aim of this study is to establish precise and identifiable classifications for quantum bioinformatics. Achieving a comprehensive classification requires careful consideration of the data types, tools, and computational operations involved in quantum bioinformatics (refer to Table 1). The presented classification relies on three primary factors. The first factor pertains to ‘The Nature of Computational Operations,’ which delineates the character and variety of computational operations employed. This aspect is typically categorized into two main groups: one involving calculations and tools for data mining processes, and the other encompassing the design, modeling, or construction of computational tools or optimization. The second factor is the ‘Type of Platform,’ which delineates the kind of computing device utilized for these calculations, encompassing classical and quantum computers. The third factor pertains to the ‘Type of Biological Data’ employed in these calculations, distinguishing between quantum biological data and classical biological data. By thoroughly analyzing these factors, we can develop a comprehensive classification system that enhances our understanding of quantum bioinformatics and its practical applications.

**Table 1:** Classification in quantum bioinformatics. A quantum biological data set is a collection of information obtained by using quantum physics tools on biological samples. A classical biological data set include a variety of types of information, including genomic, proteomic, and metabolic information. * Quantum computer-based research. † Classical computer-based research. # Quantum biological data-based research. $ Classical biological data-based research. Application of QCt-bioinformatics tools on the classical computer platform is classified as classical bioinformatics

Title of category	Abbreviation	The Nature of Computational Operations	Type of Platform	Type of Data	Related Research
Quantum Bioinformatics	Q-bioinformatics or Q-Bioinformatics or Q-B	Designing, modeling or building computational tools or optimization using quantum concepts	Classical computer or Quantum computer	Quantum Biological Data	†# DOI: 10.1016/j.biosystems.2020.104340 [173] †# DOI: 10.1016/j.jtbi.2015.11.018 [174] †# DOI: 10.1038/srep00571 [175] *# DOI: 10.1038/s41534-021-00368-4 [176] *# DOI: 10.1016/j.patter.2023.100705 [177] *# DOI: 10.1088/1751-8121/ab0313 [178]
Quantum Concept in Bioinformatics	QCt-bioinformatics or QCt-Bioinformatics or QCt-B	Designing, modeling or building computational tools or optimization using quantum concepts	Classical computer or Quantum computer	Classical Biological Data	†$ DOI: 10.1088/2058-9565/ac4f2f [179] †$ DOI: 10.1371/journal.pcbi.1011033 [180] *$ DOI: 10.1371/journal.pone.0249850 [181] *$ DOI: 10.3390/electronics10192433 [182] *$ DOI: 10.1016/j.pbiomolbio.2017.05.013 [183] *$ DOI: 10.1038/s41598-021-88,321-5 [184]
Quantum Computing in Bioinformatics	QCg-bioinformatics or QCg-Bioinformatics or QCg-B	Application of Q-bioinformatics tools in analyses	Classical computer or Quantum computer	Quantum Biological Data	*# DOI: 10.1002/qua.26975 [185] †# DOI: 10.1038/s41598-019-43,697-3 [186] †# DOI: 10.1103/PhysRevE.75.031919 [187]
Quantum Computer in Bioinformatics	QCr-bioinformatics or QCr-Bioinformatics or QCr-B	Application of classical bioinformatics tools or QCt-bioinformatics tools in analyses	Quantum computer	Classical or Quantum Biological Data	*# DOI: 10.1093/bioinformatics/btac789 [188] *$ DOI: 01. 10.1371/journal.pone.0259101 [189] *$ DOI: 10.1186/s12859-022-04661-7 [190]

### Quantum bioinformatics (Q-B)

Our research determined a subfield of bioinformatics called quantum bioinformatics, or Q-bioinformatics/Q-Bioinformatics (Q-B for short), which involves the development of computational tools using quantum biology data. The main aim of this field is to design, build, or model these tools to improve our understanding of biological systems [5]. Both quantum computers and classical computers can be used to perform these computing operations.

One crucial aspect of Q-B is its focus on data with a purely quantum nature, which is obtained from laboratory tools used in quantum mechanics. This data is distinct from classical biological data, such as genomic, proteomic, and metabolic data, which are not utilized in this field [183]. As a result, Q-B is a unique and specialized field that requires a deep understanding of both quantum mechanics and biology.

It is worth noting that Q-B is not exclusive to the use of quantum computers, and classical computers can also be utilized to carry out computational operations in this field. This flexibility allows researchers to choose the appropriate computational resources depending on the nature of the problem and the available resources.

This field has witnessed a multitude of research endeavors, collectively illuminating the profound potential of Q-B to enhance our comprehension of biological systems [173–178]. Table 1 provides a glimpse of selected exemplar studies in this context, underscoring the diversity of applications within the purview of Q-B and underscoring the pivotal role of this discipline in advancing our understanding of intricate biological systems.

One notable study delves into the intricate mechanisms by which proteins embark on targeted searches for specific DNA loci, proposing a quantum-based mechanism. This investigation introduces the innovative concepts of π-π entanglement and Quantum Walk (QW) to elucidate the dynamics of protein-DNA interactions. Notably, the exploration delves into the quantum attributes governing phenomena like walker quadratic spreading in QW, shedding light on their contributions to the expeditiousness of protein searches. It is noteworthy that this study employed quantum biological data and was conducted using a classical computer platform [173].

Furthermore, another scholarly inquiry presents a quantum mechanical model to unravel the enigmatic process through which type II endonucleases trigger DNA double-strand breaks. Within this conceptual framework, the study posits the involvement of quantum phenomena, including coherent oscillations and quantum shielding, in orchestrating this intricate phenomenon. The model incorporates considerations of symmetry and quantum entanglement as integral aspects of DNA behavior. It is important to note that this study relied on quantum biological data and was conducted using a classical computer platform [174].

Additionally, a separate exploration delves into the realm of quantum annealing and its application to lattice protein folding models, encompassing scenarios characterized by interactions among all 20 amino acids. The study meticulously compares quantum annealing with classical simulated annealing, particularly in the context of biophysical challenges. This pioneering effort paves the way for the exploration of optimization problems within the domains of biophysics and statistical mechanics through the adept utilization of quantum devices. It is pertinent to mention that this study utilized quantum biological data and was conducted using a classical computer platform [175].

Moreover, an investigation addresses the formidable challenge of predicting protein structures from the primary sequences of amino acids, a task colloquially referred to as protein folding. To tackle this complex problem, the study introduces quantum variational algorithms tailored to the folding of polymer chains through quantum computing. The research effectively demonstrates the feasibility of employing quantum algorithms on tangible quantum processors, showcasing their potential in deciphering intricate biological conundrums. Importantly, this study was conducted using quantum biological data and the quantum computer platform [176].

In parallel, another scholarly discourse navigates the domain of systems biology, exploring the application of quantum computing to logic-based models. The study underscores the daunting issue of exponential complexity growth encountered by logic-based models, advocating for the adoption of quantum computing as a potent antidote to alleviate complexity while elevating the fidelity of simulations. A specific case study pertaining to mammalian cortical development illustrates the practical applicability of this approach. It is pertinent to mention that this study was conducted using quantum biological data and the quantum computer platform [177].

Furthermore, an inquiry delves into the utility of quantum channel simulations in modeling biological multi-taxa within the realm of phylogenetics. Quantum channels serve as the foundation for constructing simulations encompassing a diverse array of phylogenetic branching models across distinct tree topologies. The primary focus centers on the emulation of statistical inference pertaining to biological attributes through the adept deployment of quantum techniques. The study also introduces the concept of a quantum pruning map as a means to enhance modeling precision. Importantly, this study was conducted using quantum biological data and the quantum computer platform [178].

Together, these inquiries emphasize the diverse array of areas where quantum computing and quantum mechanics intersect with biology, spanning domains such as phylogenetics, systems biology, protein folding, DNA interactions, and protein-DNA search mechanisms. These investigations reside within the domain of Q-B, encompassing activities related to the design, modeling, or creation of computational tools and optimization techniques rooted in quantum principles, and their application to quantum biological data. These pursuits may utilize classical or quantum computing platforms, but they all belong to the realm of Q-B.

The essential difference between Q-B data and quantum biology data mining and analysis lies in their focus and computational processes. Quantum biology primarily involves the extraction of quantum data from biological sources using laboratory tools rooted in quantum mechanics and quantum chemistry. Its primary objective is to unravel the fundamental principles of quantum mechanics within biological systems. On the other hand, quantum bioinformatics specifically concentrates on the utilization of quantum computing methods for the analysis of biological data. Quantum bioinformatics involves the development, modeling, and designing of computational tools and optimization techniques that leverage quantum principles for various biological purposes. The distinction is marked by the source of data and the computational methods employed: quantum biology deals with extracting data from biological sources using quantum tools, while quantum bioinformatics uses quantum computing for the analysis of biological data.

### Quantum concept in bioinformatics (QCt-B)

This research has unveiled an emerging area within the field of bioinformatics called Quantum Concept in Bioinformatics, abbreviated as QCt-bioinformatics/QCt-Bioinformatics (QCt-B). This subfield aims to create computational tools that harness the principles of quantum mechanics. These tools leverage concepts such as parallelism, superposition, and entanglement from quantum mechanics to analyze classical biological data, including genomic and proteomic information.

The development of these tools encompasses the formulation of diverse algorithms, with quantum parallelism serving as a foundational principle in their design. An essential characteristic of QCt-B lies in its dynamic nature, encompassing the design, construction, and modeling of computational tools. It is important to note that QCt-B is not solely reliant on quantum computers; classical computers can also execute the computational operations in this field. As a result of this flexibility, researchers are able to choose the optimal computational resources depending on the nature of the problem and the available resources.

Numerous research studies have delved into the potential of QCt-B to enhance our understanding of complex biological systems [179–184]. Table 1 provides an overview of select studies exemplifying the diverse applications of QCt-B in advancing our comprehension of intricate biological systems.

One study introduces a quantum-based approach to tackle protein design challenges. Protein design entails rearranging amino acids within sequences to create novel functions. Conventional methods face limitations due to their exponential nature. To overcome this constraint, the study presents quantum circuits employing Grover’s algorithm. These circuits accommodate customizable energy tables and protein structure models. Practicality is verified through quantum computer simulations, showcasing a quadratic speedup over classical methods. Notably, this study leveraged classical biological data and a Classical computer platform [180].

Another study focuses on reference-free DNA sequence reconstruction using quantum computation. This represents the pioneering application of quantum computing in bioinformatics. The implementation encompasses both gate-based and quantum annealing platforms. The study discusses results and limitations pertaining to classical simulations and available quantum hardware. It is important to note that this study relied on classical biological data and a quantum computer platform [181].

Yet another study addresses genome sequence reconstruction using small-scale quantum processors. Quantum algorithms are explored for sub-sequence alignment, with the QiBAM algorithm extending Grover’s search capabilities to handle approximate pattern matching in the presence of read errors. Additionally, it enables distributed searches for multiple solutions within quantum-encoded DNA sequences. This approach offers a quadratic speedup in comparison to classical methods. A comprehensive implementation is provided and verified using quantum tools such as the OpenQL compiler and QX Simulator. Similar to the previous studies, this research utilized classical biological data and a quantum computer platform [182].

Collectively, these studies underscore the growing influence of quantum computing in bioinformatics and genomics, presenting promising solutions to intricate challenges in protein design and DNA sequence reconstruction. These studies fall within the purview of QCt-B, involving the design, modeling, or development of computational tools or optimization methods based on quantum principles, applied to classical biological data. These endeavors may employ either classical or quantum computer platforms, but they are all categorized within the field of QCt-B.

### Quantum computing in bioinformatics (QCg-B)

This research has uncovered an intriguing subfield of bioinformatics called Quantum Computing in Bioinformatics, abbreviated as QCg-bioinformatics/QCg-Bioinformatics (QCg-B). The primary objective of this field is to employ quantum Bioinformatics (Q-B) computational tools for the analysis of quantum biology data and enhance our comprehension of biological systems. In contrast to conventional bioinformatics, which primarily focuses on classical biological data such as genomics, proteomics, and metabolomics, QCg-B places special emphasis on quantum biology data obtained from laboratory tools employed in quantum mechanics.

One noteworthy aspect of QCg-B is its flexibility, allowing researchers without access to quantum computers to utilize available resources and address problems based on their nature. It is important to note that QCg-B is not exclusively reliant on quantum computers; classical computers can also perform computational operations in this field. Nonetheless, this flexibility empowers researchers without access to quantum computers to utilize existing resources and solve problems according to their specific requirements.

Despite the current absence of quantum computers, a growing body of research has delved into the realm of QCg-B, demonstrating its potential to revolutionize the field [185–187]. Table 1 provides illustrative examples of these studies, spotlighting the diverse applications of QCg-B that underscore its pivotal role in advancing our comprehension of intricate biological systems.

In one study, a novel computational workflow is introduced, amalgamating classical and quantum methodologies for the study of protein-ligand interactions. This innovative workflow seamlessly integrates Density Matrix Embedding Theory (DMET) with the Variational Quantum Eigensolver (VQE) to ascertain molecular electronic ground states. Real quantum computers, encompassing superconducting transmon and trapped-ion devices, are harnessed to rank-order a series of -secretase (BACE1) inhibitors based on binding energy disparities. Notably, this marks the pioneering application of authentic quantum computers in the computation of protein-ligand binding energies. The ensuing findings offer valuable insights into the hardware and software prerequisites necessary for the effective implementation of Noisy Intermediate Scale Quantum (NISQ) algorithms in the realm of drug design. It’s important to highlight that this research draws upon quantum biological data and leverages a quantum computer platform [185].

Another study ventures into the domain of quantum pattern recognition techniques to address the burgeoning challenge of processing massive sequence databases generated by high-throughput sequencing technologies. The escalating volume of sequence data necessitates the development of more efficient methods while upholding data accuracy. This study pioneers a sophisticated approach that combines the conventional dot-plot method with quantum algorithms, augmenting pairwise sequence alignment. Quantum parallelism and insights from diffraction patterns of X-rays converge to formulate an ingenious array indexing structure for managing expanding sequence databases. Comparative assessments vis-à-vis classical aligners underscore the method’s prowess, showcasing superior alignment quality and heightened efficiency in terms of time and space complexity. Remarkably, this research harnesses quantum biological data while employing a classical computer platform [186].

Both research serve as compelling testaments to the potential of quantum computing in tackling intricate challenges within the realm of bioinformatics. The first article meticulously explores the quantification of protein-ligand interactions, employing a hybrid strategy that bridges classical and quantum methodologies, thus offering invaluable insights into the practical deployment of quantum algorithms in drug design. The second article navigates the uncharted waters of quantum pattern recognition to enhance sequence alignment, ushering in enhanced efficiency and precision when compared to conventional methods. These endeavors are firmly entrenched within the domain of QCg-B, which encompasses the application of Q-B tools in analyzing classical biological data. While these undertakings may exploit both classical or quantum computer platforms, they undoubtedly find their home in the realm of QCg-B. As QCg-B evolves, it harbors the promise of catalyzing groundbreaking advancements in the field of bioinformatics, fundamentally reshaping our approach to biological research.

### Quantum Computer in Bioinformatics (QCr-B)

This research has uncovered a subfield of bioinformatics known as Quantum Computer in Bioinformatics, abbreviated as QCr-bioinformatics/QCr-Bioinformatics (QCr-B). In this field, classical bioinformatics computational tools and QCt-bioinformatics tools are used to analyze both classical biological data (such as genomic, proteomic, and metabolic data) and quantum biology data (which is obtained using quantum physics tools on biological samples) on high-performance computing platform, such as a quantum computer.

Unlike QCg-B, which focuses exclusively on quantum data and employs either quantum or classical computers for computational operations, QCr-B leverages quantum computers solely for their speed and storage capacity while utilizing bioinformatics computational tools and QCt-bioinformatics tools or classical biological and quantum biology data in its analysis.

This distinctive approach to bioinformatics presents exciting opportunities for researchers to harness the immense power of quantum computers in tackling complex biological problems. By taking advantage of the speed and storage capabilities of quantum computers, QCr-B has the potential to significantly advance our understanding of biological systems.

It is important to note that the computing operations in QCr-B rely on classical bioinformatics computational tools or QCt-bioinformatics tools. This provides flexibility for researchers who have access to quantum computers, as they can utilize available resources to solve problems according to their specific requirements.

Several research studies have already been conducted in this field, demonstrating the potential of QCr-B to enhance our understanding of biological systems [188–190]. Table 1 presents some examples of these studies, showcasing a range of potential applications of QCr-B and underscoring the importance of this field in advancing our knowledge of complex biological systems.

## CONCLUSION

In conclusion, the fields of bioinformatics, quantum biology, and quantum bioinformatics share a common objective of analyzing and interpreting biological data to gain a comprehensive understanding of biological processes. However, the diverse computational processes employed in each field present several challenges within the realm of quantum bioinformatics. These challenges encompass the ambiguity surrounding the definitions of quantum biology and quantum bioinformatics, the complexities of isolating purely quantum effects from other factors, and the necessity to establish classifications for distinct subsets of quantum bioinformatics.

To address these challenges effectively, it is imperative to establish precise and unambiguous definitions for quantum biology and quantum bioinformatics while fostering the development of standardized terminology within the field. Furthermore, the formulation of a comprehensive classification scheme is indispensable to categorize the various types of quantum data that can be obtained from scientific instruments. This review has provided a classification scheme based on the types of quantum data acquired from diverse instruments, encompassing quantum state data, quantum measurement data, quantum communication data, quantum computing data, quantum sensing data, and quantum imaging data.

By presenting a specific and identifiable classification scheme for quantum bioinformatics, this study has contributed to the attainment of a more comprehensive understanding of the field. This classification has been achieved through a nuanced understanding of computational operations, computing platforms, and data types. Notably, it highlights that the utilization of a quantum computer is only necessary within a specific subset of quantum bioinformatics, enabling researchers to engage in valuable research even in the absence of direct access to a quantum computer.

Future research endeavors can build upon this classification scheme to further propel the advancement of quantum bioinformatics. Ultimately, the progress made in quantum bioinformatics will empower scientists to delve deeper into the intricacies of biological processes and drive the development of innovative treatments and therapies for a wide range of diseases.

## KEY POINTS

In this study, our aim was to explore the integration of quantum mechanics with the realms of biology and bioinformatics. Moreover, our objective was to clarify the fundamental concepts within quantum biology and quantum bioinformatics, establishing clear demarcations to prevent confusion among researchers and encourage interdisciplinary collaborations. Finally, we introduce a taxonomy for quantum bioinformatics, organized according to the types of computational operations, platforms utilized, and data categories involved. This classification comprises four classes: Q-Bioinformatics, QCt-Bioinformatics, QCg-Bioinformatics, and QCr-Bioinformatics.

**Key Points**

- Quantum bioinformatics faces challenges requiring precise definitions, standardized terms, and a thorough classification system. The article introduces a system categorizing based on computational operations, platform type, and biological data.
- Q-Bioinformatics (Q-B) specializes in the development of computational tools that exclusively utilize quantum biological data derived from biological sources.
- QCt-Bioinformatics (QCt-B) entails the creation of tools utilizing quantum mechanics principles to scrutinize classical biological data.
- QCg-Bioinformatics (QCg-B) employs Q-B tools to analyze quantum biological data, demonstrating flexibility across both quantum and classical computers.
- QCr-Bioinformatics (QCr-B) uses quantum computers solely for speed and storage while applying classical bioinformatics tools.

## Data Availability

This article has no additional data.
